# Intrauterine infusion of clinically graded human umbilical cord-derived mesenchymal stem cells for the treatment of poor healing after uterine injury: a phase I clinical trial

**DOI:** 10.1186/s13287-022-02756-9

**Published:** 2022-03-03

**Authors:** Jingrui Huang, Qi Li, Xiaohua Yuan, Qiaoshu Liu, Weishe Zhang, Ping Li

**Affiliations:** 1 Department of Obstetrics, Xiangya Hospital, Central South University, Changsha, 410008 Hunan People’s Republic of China; 2 Hunan Engineering Research Center of Early Life Development and Disease Prevention, Changsha, 410008 Hunan People’s Republic of China; 3 Reproductive Medicine Center, Xiangya Hospital, Central South University, Changsha, 410008 Hunan People’s Republic of China

**Keywords:** huUC-MSCs, Poor healing after uterus injury, Safety, Efficacy, Clinical translation

## Abstract

**Background:**

Intrauterine adhesion and cesarean scar diverticulum are the main complications of poor healing after uterine injury. Human umbilical cord MSCs transplantation has been regarded as the most potential treatment in the clinic, the safety and efficacy of which in the clinic, however, remains unclear.

**Methods:**

In this study, ten patients were enrolled: six with intrauterine adhesion and four with cesarean scar diverticulum. All the patients were injected with human umbilical cord MSCs twice into the uterus. Beside the chest X-ray, ECG and abdominal ultrasound, many laboratory tests including blood routine, liver and renal function, ovarian function, tumor biomarkers, and immune function were used to estimate the safe after stem cell transplanted. In addition, the efficacy of stem cell transplanted was shown by the endometrial thickness, the volume of the uterus, and cesarean scar diverticulum based on 3D ultrasound imaging.

**Results:**

We found that all results of these laboratory tests were normal in these enrolled patients before and after cell injection. Meanwhile, the results of the chest X-ray and ECG were also normal in the treatment process. The abdominal ultrasound showed that the size of the left and right kidneys was inconsistent in one patient after cell therapy, while those of other patients were normal. In addition, endometrial thickness, the volume of the uterus, and cesarean scar diverticulum showed an improving tendency, but no significant difference was noted.

**Conclusion:**

In summary, intrauterine injection of clinically graded human umbilical cord MSCs was safe for poor healing after uterus injury.

*Trial registration* NCT03386708. Registered 27 December 2017, https://clinicaltrials.gov/ct2/show/NCT03386708?cond=CSD&cntry=CN&draw=2&rank=2

**Supplementary Information:**

The online version contains supplementary material available at 10.1186/s13287-022-02756-9.

## Background

The poor healing after uterine injury mainly includes intrauterine adhesion and cesarean scar diverticulum. Patients subsequently often suffer from amenorrhea, polyhypomenorrhea, prolonged menstrual periods, or infertility [1, 2]. The poor healing of uterine injury has attracted more attention of researchers with the increasing rate of cesarean section and abortion. The common treatments currently available are drug treatment and hysteroscopic surgery [2, 3], which, unfortunately, have not been proven to be completely effective with some side effects, such as infections, thrombosis, and liver function damage. Therefore, effective and safe treatment for those who respond poorly to current therapies is demanding.

In recent years, mesenchymal stem cells (MSCs) have been widely used to treat various tissue and organ damages [4–10], which are derived from various tissues including bone marrow, umbilical cord, placenta, and cord blood with multipotent mesodermal differentiation potential. More importantly, they have been found to secrete various cytokines and trophic factors, with strong anti-inflammatory and immunomodulatory properties that promote tissue repair [11, 12]. And the umbilical cord derived from the discarded medical tissue has caught the eyes of researchers for its high in vitro proliferation rate [13]. Meanwhile, it is also the main source in regenerative medicine for its easy extraction, noninvasiveness, and no ethical constraints.

Some studies and case reports have shown that the injection of autologous stem cells (from bone marrow, peripheral blood, or menstrual blood) can improve endometrium regeneration [14–17]. However, the different quantity and quality of autologous stem cells among patients with different injection methods and times influence the therapeutic outcomes of patients. In addition, the efficacy of MSCs transplanted in cesarean scar diverticulum and intrauterine adhesion without surgical treatment remains unclear. Herein, a novel therapy using clinical-grade human UC-MSCs was proposed to treat the patients with poor healing after uterine injury in this study, and the results of the phase I clinical trial were presented.

## Methods

### Patient population

Adult women (18–40 years) with poor healing after uterine injury were enrolled according to the results of ultrasound or hysteroscopy. The inclusion criteria were as follows: patients with BMI ranging from 18 to 24 kg/m^2^ who suffer from poor healing after uterine injury with the symptoms of cesarean scar diverticulum and intrauterine adhesion and respond poorly to conventional treatment. The exclusion criteria were as follows: patients in pregnancy or breastfeeding, patients with severe medical and surgical complications or an active infection, patients with a history of incompletely treated tuberculosis and cervical lesions, and patients with other cell products injection in the past six months.

### Study design

The present study was performed between December 2019 and December 2020 at Xiangya hospital Central South University in China. The protocol was approved by the stem cell clinical research academic and ethics committee of Xiangya hospital Central South University (Ethics number: 201708002) and China Food and Drug Administration. This is a no-randomized, no-blinded study for patients with poor healing after uterine injury (ClinicalTrials.gov NCT03386708). All patients signed informed consent.

### MSC preparation

Human umbilical cords were obtained from a full-term cesarean section surgery at Xiangya Hospital (Changsha, China). The patients had been informed in advance and consented to donate. The clinically graded human umbilical cord MSCs were isolated and cultured in the SCLNOW Corporation (Beijing, China). The detailed procedure for obtaining MSCs was as follows: Human umbilical cord, from cesarean delivery, immersed in a decontaminating solution, were transported to sterile laboratory by the cold chain transportation within 6 h. The outer amniotic membrane and Wharton's jelly were separated and minced to 1 mm^3^ pieces after washing with D-Hank’s buffer. An optimized precise proportion among tissue mass, enzyme activity units, digestion solution volume, and void volume was used for the isolation of cells from the umbilical cord tissue. And the serum-free stepwise culture process was followed growth medium (iSCLCODE^®^). UC-MSCs were plated in culture flask with serum-free medium and placed in a humidified incubator at 37 °C, 5% CO2 to obtain enough numbers before mixed with support material iECM^®^ hydrogel purified from Wharton’s jelly at protein concentration of 1 mg/ml. Cells from passages (P) 5 were used for following experiments; then, the osteogenesis and adipogenesis experiments, growth curves, the expression of pluripotent genes and flow cytometry analysis of umbilical cord MSCs, are carried out to verify it (detailed in Additional files 1, 2). All the clinical-graded human umbilical cord MSCs had passed the quality testing by the General Administration of Quality Supervision, Inspection and Quarantine of the People's Republic of China. On the day of injection, the stem cell (2*10^7^) was sent to Xiangya hospital (Changsha Chian) by airplane, where the officers from the stem cell laboratory in the hospital would re-examine the quality of stem cell. The stem cells will be used in clinical transplant if their viability is greater than 85%.

### Intrauterine injection

Two experienced obstetricians performed the intrauterine injection under ultrasound guidance. Firstly, the operator scraped the lesion tissue with the little spatula until the depth of the uterine cavity was greater than 6 cm. Then an 18F Foley catheter was placed into the uterine cavity, followed by the injection of 1 ml saline into the catheter bulb to block the cervix. Then 2 ml (1*10^7^/ml) of clinical-grade umbilical cord MSCs was spread onto an 18F Foley catheter and placed into the uterine cavity. Finally, the catheter was removed after withdrawing saline in the bulb after 24 h. The procedure was performed on the tenth day of the menstrual period.

### Follow-up

#### Adverse events and safety assessment

All complaints of discomfort were recorded in this study. The results of blood routine (white blood cell count, hemoglobin, platelets), ovarian function assessment (Anti-Mullerian hormone, AMH), liver function assessment (ALT, AST, total bilirubin, direct bilirubin, indirect bilirubin, total bile acid, and albumin), renal function assessment (Creatinine, Urea Nitrogen, Urine acid), immune function (complement C3/C4 and IgG, IgA, IgM), tumor biomarker (C12), abdominal ultrasound (digestive system and urinary system), ECG, and chest X-ray were recorded.

### Efficacy assessment

#### Patient-reported outcomes

The volume of menstruation was recorded by all patients according to a pictorial chart [18] before and after cell transplanted, so was the length of menstruation.

### Transvaginal ultrasound

Baseline (during the natural menstrual cycle before cell transplanted) and follow-up (3 and 6 months after cell transplanted) endometrial thickness, the volume of cesarean scar diverticulum and uterus were measured by a Doppler ultrasound scan (Philips EPIQ7 Ultrasound system, Royal Dutch Philips Electronics Ltd., Netherlands) to evaluate rehabilitation situation after uterine injury. And the 3D imaging system from the Philips EPIQ7 ultrasound system was also used to 3D reconstruct the shape of the cesarean scar diverticulum for quantitative analysis of the volume of the cesarean scar diverticulum and uterus.

### Statistical analysis

Continuous variables in the clinical data were presented as means and standard deviations. Discrete variables were reported as frequencies and proportions. The data for the safety evaluation before and after the treatment were compared by ANOVA. The statistical significance of the analyses was determined by a two-tailed *P* value of < 0.05.

## Results

### Participant flow

**Fig. 1:**
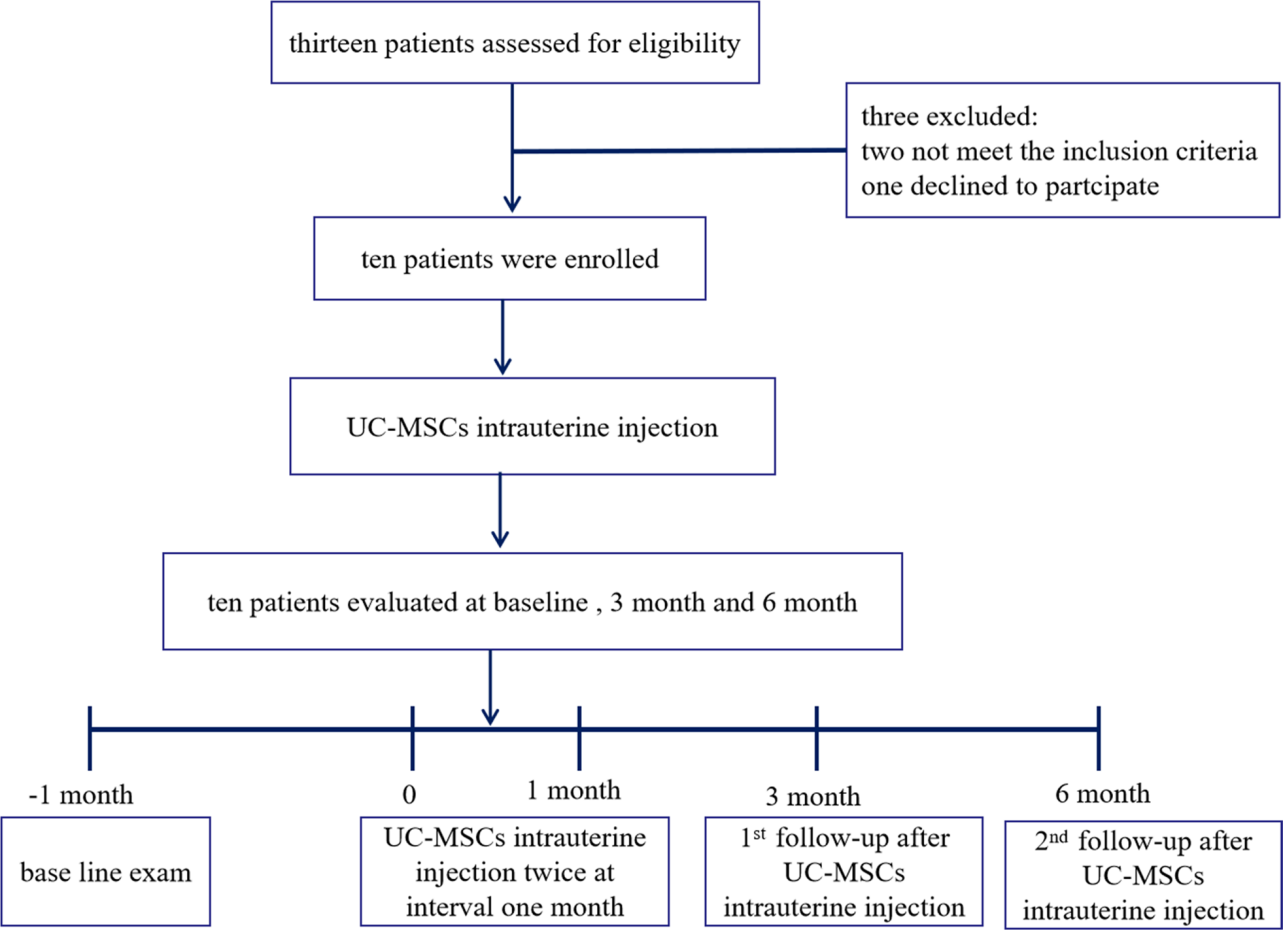
Study flow diagram

Ten patients with poor healing after uterine injury: six with intrauterine adhesion and four with cesarean scar diverticulum, were assessed over 6 months following the steps outlined in the flowchart (Fig. 1).

### Baseline characteristics of the patients

All ten patients had experienced at least one hysteroscope and drug treatment that had been confirmed invalid. The average diagnosis duration ranged from 1 to 6 years after the uterine injury. The clinical characteristics of the patients enrolled in the study are shown in Table 1. The average age of the patients was 33.9 ± 3.75 years old (from 29 to 40 years old) at the time of intervention. Four patients had the symptom of hypomenorrhea, two patients amenorrhea, and four prolonged menstrual periods.

**Table 1:** Patients demographic and injection dosing

Study ID	Age	Disease duration (years)	Weight (kg)	Height (cm)	Diagnosis	Clinical symptoms	The dose of hUC-MSCs per IU injection	
							1st dose	2nd dose
01	30	6	55	163	CSD	Prolonged Menstrual period	1.85 × 10^7^	2.14 × 10^7^
02	38	4	51	156	IUA	Hypomenorrhea	1.89 × 10^7^	2.08 × 10^7^
03	30	2	50	163	IUA	Hypomenorrhea	1.90 × 10^7^	1.94 × 10^7^
04	29	4	57	158	IUA	Amenorrhea	1.84 × 10^7^	1.90 × 10^7^
05	38	2	54	156	IUA	Hypomenorrhea	2.04 × 10^7^	2.18 × 10^7^
06	34	1	58	167	CSD	Prolonged Menstrual period	1.86 × 10^7^	2.12 × 10^7^
07	34	6	49	158	IUA	Hypomenorrhea	2.08 × 10^7^	1.92 × 10^7^
08	40	1	54	165	CSD	Prolonged Menstrual period	2.02 × 10^7^	1.91 × 10^7^
09	33	2	52	152	CSD	Prolonged Menstrual period	2.02 × 10^7^	2.34 × 10^7^
10	33	3	45	160	IUA	Amenorrhea	2.16 × 10^7^	2.08 × 10^7^

*CSD* cesarean scar diverticulum, *IUA* intrauterine adhesion, *hUC-MSCs* human umbilical cord mesenchymal stem cells

### Adverse events and safety assessment

In order to assess the safety of the treatment, we recorded the systemic and local safety after the cell therapy. No discomfort symptoms were reported in the enrolled ten patients. Meanwhile, the results of ECG and chest X-ray did not change before and after the cell transplantation. However, the result of abdominal ultrasound of the NO.1 patient indicated that the right kidney was smaller than the left kidney in the sixth month after cell transplantation. Fortunately, the patient did not have any uncomfortable symptoms and the results of renal function and urine routine were normal. Similar positive results did not exist in other patients. In addition, no positive results existed in the laboratory tests at any time of the follow-up period. And the means of white blood cell, hemoglobin, and platelets counts were normal before and 3 and 6 months after cell transplantation (Table 2). The assessment indicators of liver, kidney and ovarian function fluctuated within the normal range before and 3 and 6 months after cell therapy (Table 3). Meanwhile, the tumor biomarkers and immune indicators were detected at above-mentioned times, and no abnormal indicators were found (Tables 4, 5). Therefore, no adverse events occurred throughout the observation period.

**Table 2:** Quantitative analysis of blood routine at baseline and third and sixth month after cell injection

	Baseline	Third month	Sixth month	*P* value
WBC × 10^9^/L	5.88 ± 1.08	5.39 ± 1.34	4.81 ± 1.45	> 0.05
Hb g/L	130.2 ± 12.15	128.7 ± 12.49	129.4 ± 12.89	> 0.05
PLT × 10^9^/L	234.2 ± 64.17	235.8 ± 55.51	236.9 ± 60.43	> 0.05

**Table 3:** Quantitative assessment of liver, kidney, and ovarian function at baseline and third and sixth month after cell injection

	Baseline	Third month	Sixth month	*P* value
ALT U/L	14.68 ± 6.2	14.43 ± 5.29	14.2 ± 6.59	> 0.05
AST U/L	20.49 ± 5.08	19.48 ± 5.19	20.7 ± 3.75	> 0.05
BUN mmol/L	3.59 ± 0.70	4.41 ± 1.39	4.60 ± 1.68	> 0.05
Albumin g/L	45.86 ± 2.8	44.64 ± 2.27	45.31 ± 0.97	> 0.05
Creatinine μmol/L	62.93 ± 5.71	64.41 ± 5.5	65.83 ± 4.88	> 0.05
TBIL μmol/L	12.05 ± 4.18	11.37 ± 4.73	8.63 ± 2.87	> 0.05
DBIL μmol/L	6.3 ± 1.9	5.65 ± 2.48	3.98 ± 1.12	> 0.05
TBA μmol/L	2.75 ± 2.72	2.55 ± 2.44	1.47 ± 1.0	> 0.05
Uric Acid μmol/L	291.6 ± 89.21	291.6 ± 49.4	271.4 ± 63.91	> 0.05
AMH	2.75 ± 0.77	3.27 ± 1.93	4.70 ± 2.43	> 0.05

**Table 4:** Quantitative assessment of immune function at baseline and third and sixth month after cell injection

	Baseline	Third month	Sixth month	*P* value
C3 mg/L	910 ± 137.4	845.7 ± 98.99	815.8 ± 74.8	> 0.05
C4 mg/L	194 ± 65.39	176.4 ± 59.21	164.6 ± 39.23	> 0.05
Ig G mg/L	14.31 ± 3.03	14.06 ± 3.33	12.88 ± 3.72	> 0.05
Ig A mg/L	2201 ± 1381	2218 ± 1514	1899 ± 1481	> 0.05
Ig M mg/L	1536 ± 512.3	1501 ± 517	1524 ± 434	> 0.05

**Table 5:** Quantitative analysis of tumor markers at baseline and third and sixth month after cell injection

	Baseline	Third month	Sixth month	*P* value
CEA ng/ml	0.39 ± 0.34	0.49 ± 0.47	0.53 ± 0.43	> 0.05
AFP ng/ml	2.97 ± 2.01	2.35 ± 1.12	3.44 ± 1.77	> 0.05
CA 15–3 KU/L	2.34 ± 2.68	3.85 ± 3.43	6.93 ± 3.77	> 0.05
CA-199 ng/ml	8.23 ± 6.58	6.45 ± 4.58	10.46 ± 5.49	> 0.05
CA-125 U/ml	11.23 ± 7.6	12.75 ± 8.25	14.47 ± 9.63	> 0.05
CA 72–4 U/ml	1.82 ± 2.48	2.24 ± 2.89	1 ± 1.52	> 0.05
NSE ng/ml	2.25 ± 0.94	4.31 ± 2.86	6.43 ± 2.75	> 0.05

### Clinical symptoms record

Four patients with intrauterine adhesion reported that the amount of menstrual blood had been slightly increased within 3 months after cell transplanted. And then the amount of menstrual blood would be the same as the baseline at 3 months after cell therapy. The amount of menstrual blood of the other two intrauterine adhesion patients was not changed at any time after cell transplantation (Table 6). In addition, the clinical symptoms about prolonged menstrual days of four patients with cesarean scar diverticulum were not improved (Table 7).

**Table 6:**
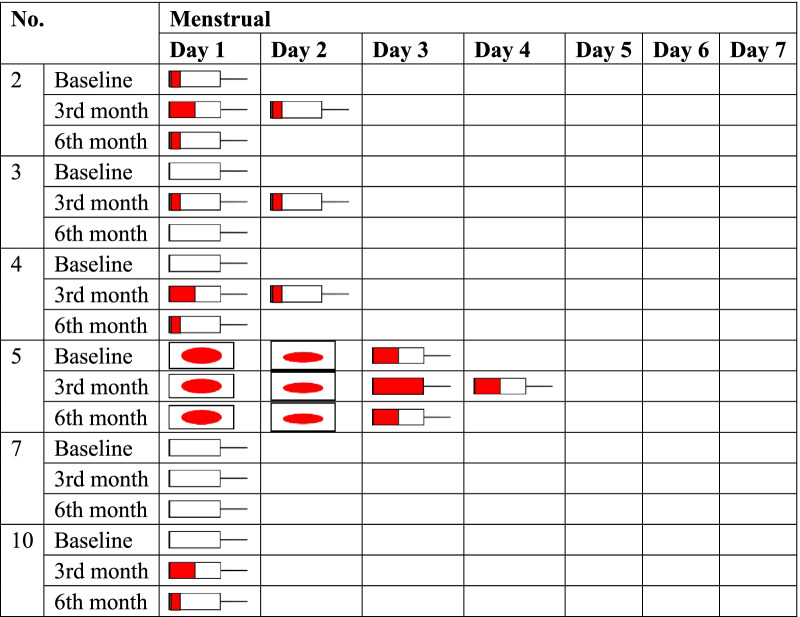
The changes of menstrual after UC-MSCs transplanted in the patients with intrauterine adhesion

**Table 7:** The changes of menstrual period after UC-MSCs transplanted in the patients with cesarean scar diverticulum (CSD)

No	Menstrual period		
	Baseline (days)	Third month (days)	Sixth month (days)
1	15	15	15
6	13	14	13
8	12	12	12
9	10	9	11

### Ultrasound scans

**Fig. 2:**
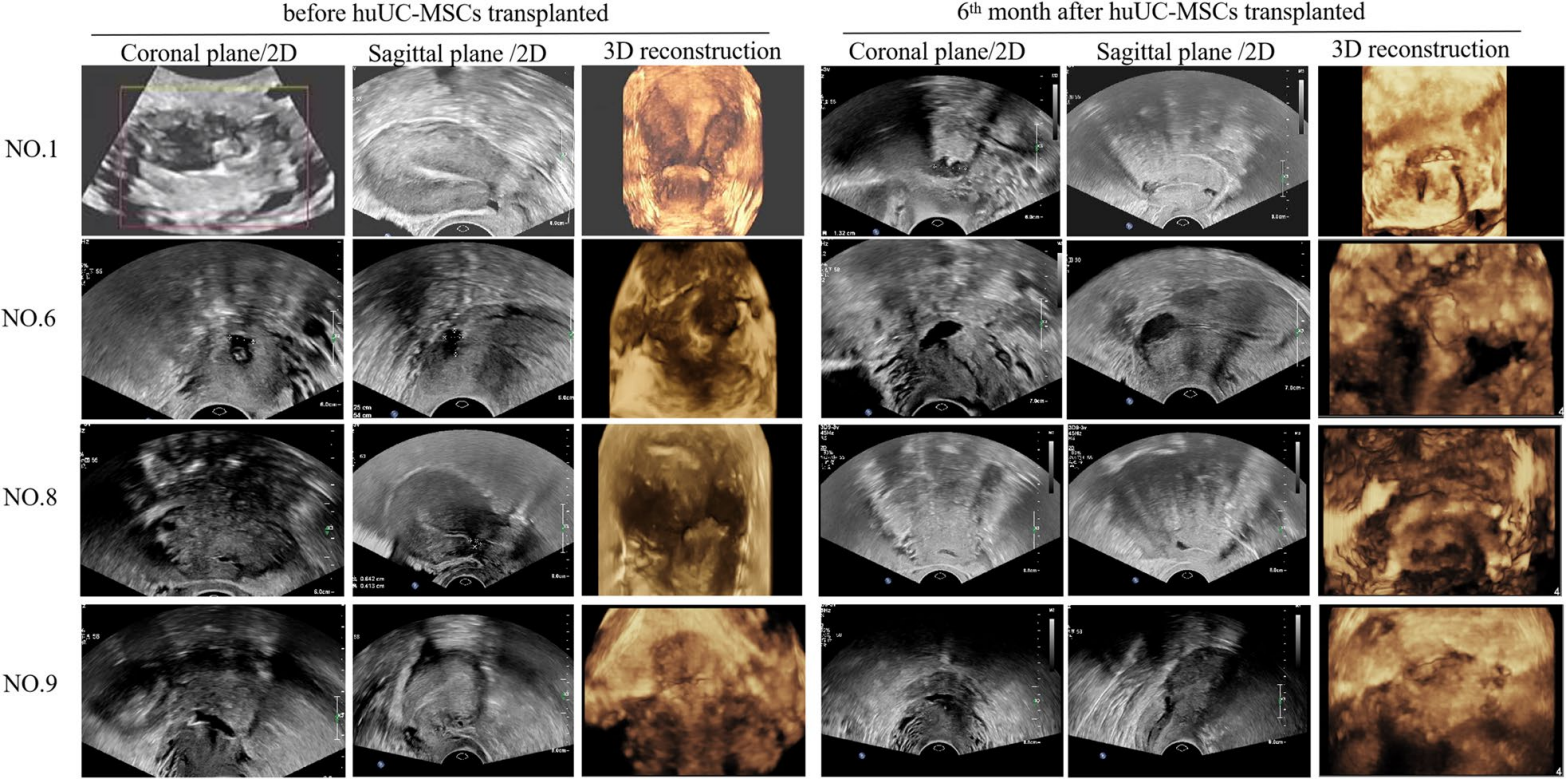
Doppler ultrasound imaging from 4 patients with cesarean scar diverticulum before and sixth month after human umbilical cord MSCs

**Fig. 3:**
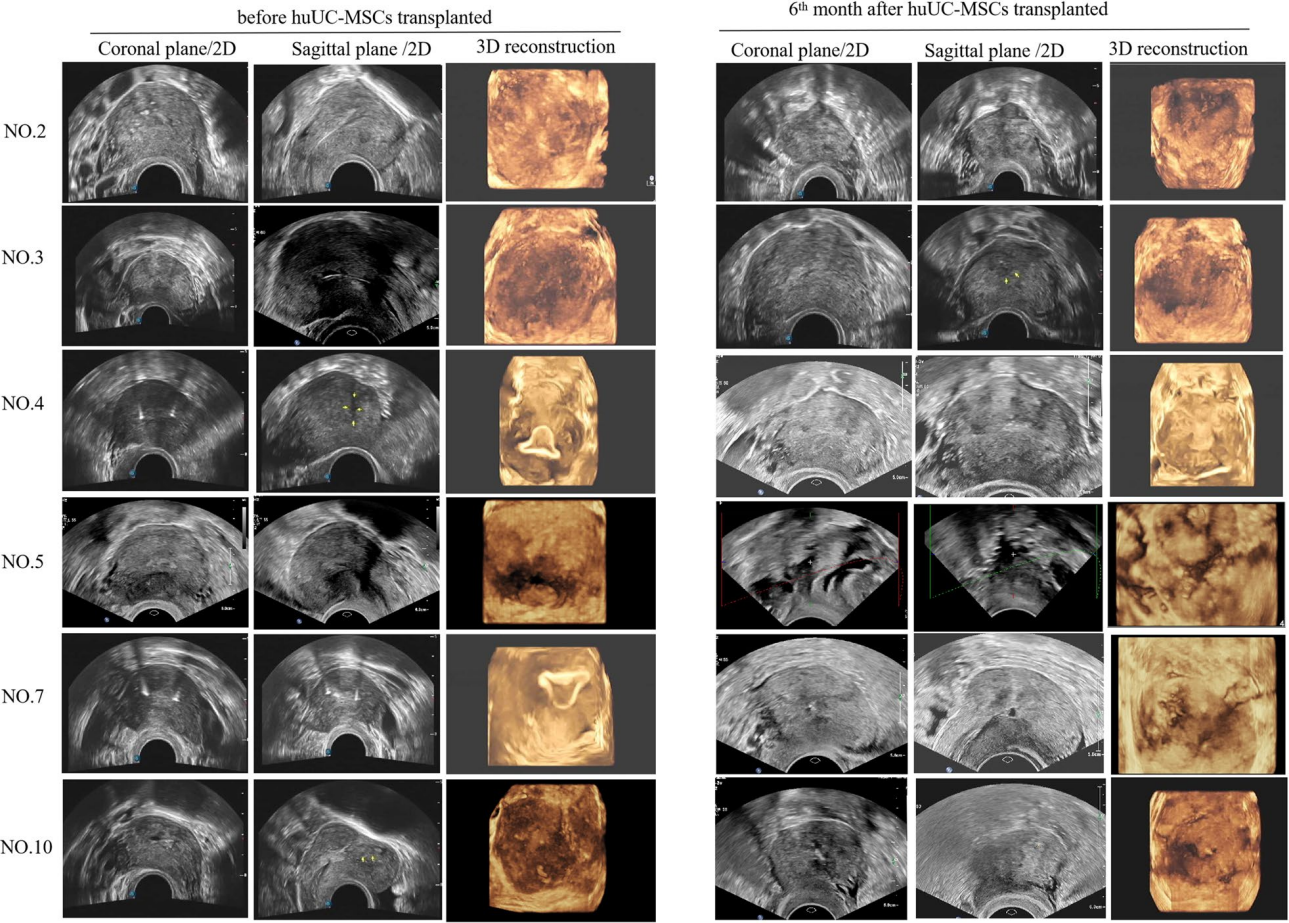
Doppler ultrasound imaging from 6 patients with intrauterine adhesion before and sixth month after human umbilical cord MSCs

**Fig. 4:**
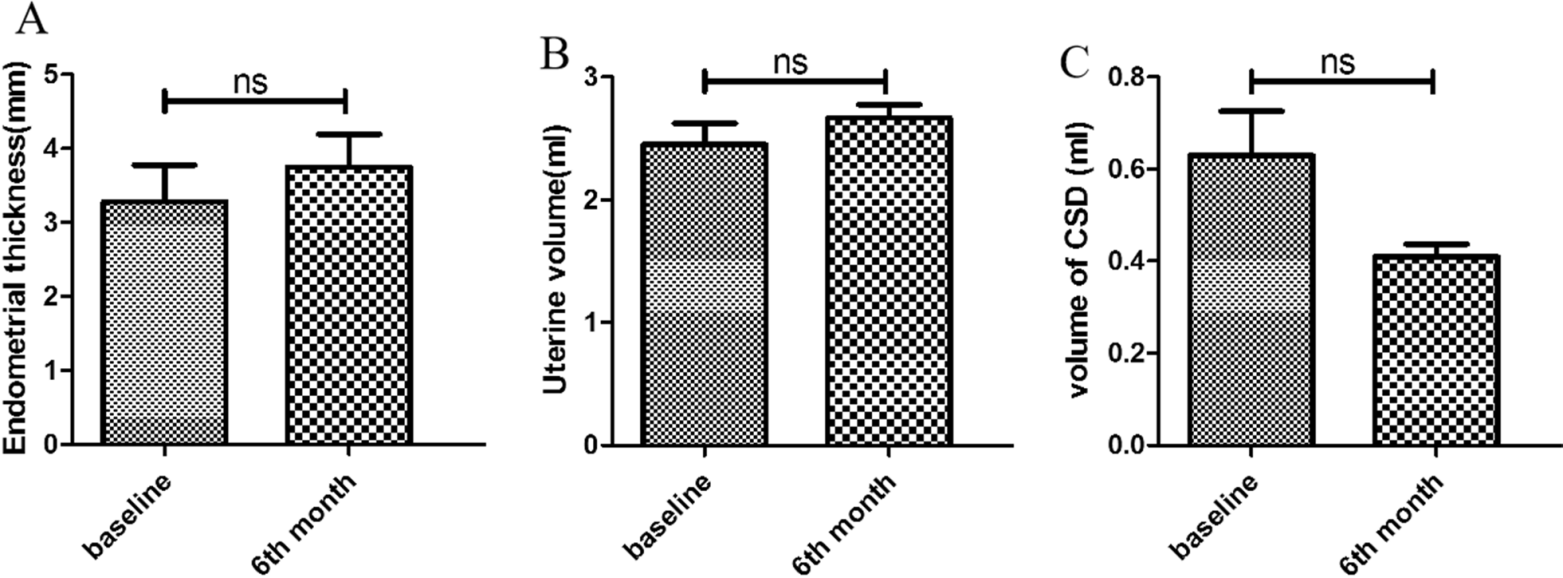
Quantitaive analysis of endometrial thickness (**A**), volume of uterus (**B**), volume of cesarean scar diverticulum (**C**)

The ultrasound results revealed the improvements tendency in the endometrial thickness, the volume of the uterine incision diverticulum, and uterine cavity at the end of the sixth month (Figs. 2, 3). Furthermore, the quantitative analysis of the above-mentioned indicators based on the ultrasound results was conducted (Fig. 4). The average maximum endometrial thickness and volume of the uterine cavity in six patients were increased from 3.28 ± 1.20 to 3.75 ± 1.08 mm and from 2.45 ± 0.42 to 2.66 ± 0.26 ml, respectively. (*P* > 0.05, Fig. 4a, b and Table 8). The average maximum volume of cesarean scar diverticulum in four patients decreased from 0.63 + 0.19 to 0.41 + 0.05 ml (*P* > 0.05, Fig. 4c and Table 9). Although the results showed an improvement tendency, there was no significant statistical difference and no long-term relief of the clinical symptoms.

**Table 8:** The efficacy assessment after UC-MSCs transplanted in the patients with intrauterine adhesion

Study no	Endometrial thickness		Uterine volume	
	Baseline	Sixth month	Baseline	Sixth month
2	4 mm	4.5 mm	2.5 ml	2.8 ml
3	2.2 mm	2.8 mm	2.8 ml	3 ml
4	5 mm	5.2 mm	3 ml	2.9 ml
5	2.2 mm	2.6 mm	1.8 ml	2.4 ml
7	4 mm	4.4 mm	2.4 ml	2.5 ml
10	2.3 mm	3 mm	2.2 ml	2.4 ml
Mean ± SD	3.28 + 1.20 mm	3.75 + 1.08 mm	2.45 + 0.42 ml	2.66 + 0.26 ml
*P* value	> 0.05		> 0.05

**Table 9:** The efficacy assessment after UC-MSCs transplanted in the patients with cesarean scar diverticulum (CSD)

Study no	Baseline	Sixth month after transplanted
	The volume of CSD	The volume of CSD
1	0.59 ml	0.45 ml
6	0.89 ml	0.46 ml
8	0.61 ml	0.38 ml
9	0.43 ml	0.35 ml
Mean ± SD	0.63 + 0.19 ml	0.41 + 0.05 ml
*P* value	> 0.05	> 0.05

## Discussion

Cell therapy has considered the most promising treatment in clinics. Mesenchymal stem cell transplantation, especially stem cells derived from the umbilical cord (UC) [19, 20] and bone marrow (BM) [21, 22], has been used in all kinds of disease models, from basic research to clinic treatment for its rich source and immune regulation function. Relative to BMSCs, UC-MSCs have the higher proliferative capacity and differentiation potential [13]. Therefore, human UC-MSCs were selected to repair the uterine injury in this study, considering their known advantages accessibility, painless acquisition, and low immunogenicity.

Studies have confirmed that MSCs derived from different sources, such as bone marrow, umbilical cord, umbilical cord blood, and menstrual blood, can promote endometrial and muscle cell proliferation and microvascular regeneration and recover the endometrial embryo-implantation ability in the animal model [23–26] and patients with uterine injury [27, 28]. However, most researchers only paid attention to the intrauterine adhesion after uterine injury, with other common complications of cesarean section like cesarean scar diverticulum ignored. According to Qiao et al. [29] in the lancet, in the past several years, the rate of cesarean section was as high as 36.7%. Correspondingly, the incidence of cesarean scar diverticulum following cesarean section is on the rise year by year. In this study, the efficacy of stem cell transplanted in cesarean scar diverticulum was firstly estimated. Meanwhile, intrauterine adhesion has the eyes of researchers with studies reporting the positive results in clinic. But in theses previous studies, the stem cell is always chosen to transplant immediately after hysteroscopy and so that some disadvantages limited its clinical transformation. For example, residual dilatation fluid occupying the uterine cavity limits the number of stem cells transplanted, and the surgery risks and costs of hysteroscopic also restrict clinical transformation. Hence, hysteroscopy was not carried out for the patients with intrauterine adhesion in this study. The results showed that clinically graded human UC-MSCs transplantation without hysteroscopic could partially release the extent of intrauterine adhesion and increase the menstrual volume and endometrial thickness in a short time.

The single injection of stem cells has been the most common treatment [30]. Recently one study [31] found good endometrial repair in the single transplantation group, but which diminished over time. In our follow-up time, it was found that the degree of improvement in clinical symptoms of the patients depended on the time after cell therapy, consistent with a previous study [31]. Furthermore, Zhang etal. [31] also found that multiple transplantations were more effective in improving rat fertility than single transplantation when treating endometrial damage. Hence, the number of stem cell transplantation was increased to observe the repair capacity after MSCs transplantation. In this study, two cell therapies were performed on enrolled patients, each transplanting 2*10^7^/ml of stem cells every time, at an interval of one menstrual cycle. Then results suggested that the safety of double stem cell transplantation was the same as that of the single transplantation.

In addition, the results showed that the clinical symptoms of the prolonged menstrual period were not released in the four patients with cesarean scar diverticulum even if the shape and volume of the cesarean scar diverticulum showed an improvement tendency. This is probably because the volume and shape of the cesarean scar diverticulum were not reduced enough to affect the improvement in clinical symptoms. Therefore, these patients may need to increase the treatment times to confirm the efficacy of cell therapy.

To ensure the safety of cell treatment, the changes in multiple systems of the body, such as heart, lung, liver, renal, ovary, blood, and immune before and after cell therapy were observed. Nine of ten patients were in the normal range of the above-mentioned indicators after cell transplantation. However, the size of both kidneys of the NO.1 patient was found inconsistent by abdominal ultrasound at the sixth month after cell therapy, while the renal function, urine routine, and blood flow of both kidneys were normal. In addition, the patient did not have any discomfortable symptoms. Then the NO.1 patient was assumed to have the congenital inconsistent development of both kidneys. Because if the intrauterine injection of UC-MSCs influenced the size of both kidneys, the stem cells had to enter the blood vessels and affect the kidneys through the circulatory system. Theoretically, If so, both kidneys would be affected, not just one. Until now, the patient is still followed up, and the size of both kidneys was the same as during the second follow-up.

Of course, there are also some limitations to this study. Firstly, the amount of stem cell transplantation should be regulated depending on the volume of the uterine, so that the appropriate dose of stem cells can be ensured while maintaining the same therapeutic effect. Secondly, the retained time of stem cells in the uterine was no more than 24 h, which would affect the therapeutic potential. Hence, clinical-grade UC-MSCs loaded onto a 3D printed scaffolds according to the volume and shape of uterine and cesarean scar diverticulum based on medical imaging will prolong the retained time after stem cell transplantation. Thirdly, it was found that the effect of stem cell therapy diminished over time. Therefore, the adequate time of stem cell transplantation will be the future plan for obtaining the best clinic effect. At last, hysteroscopy was the ideal way to directly observe the uterine cavity, but some patients refused to have a hysteroscopy in this study because they preferred the noninvasive ultrasound scanning to minimally invasive examination; therefore, only ultrasound scanning was used to detect the repair situation of uterine.

## Conclusions

In this study, the safety of two consecutive transplantations of the clinical-grade UC-MSCs into the uterine cavity without hysteroscopic surgery were observed for recurrent IUA patients and cesarean scar diverticulum. The results indicated that stem cell transplantation might be a promising treatment for patients with poor healing after uterine injury. Although some negative results were obtained on safety assessment, the efficacy of stem cell therapy will be controversial for the limited samples in the study. Therefore, the sample sizes will be enlarged to prove the efficacy of stem cell transplantation when treating patients with poor healing after uterine injury.

## Supplementary Information

**Additional file 1**. The method of isolation and quality control of clinically graded huMSCs.

**Additional file 2**. The result of isolation and quality control of clinically graded huMSCs.

## Data Availability

The data that support the findings of this study are available from Xiangya hospital Central South University, but restrictions apply to the availability of these data, which were used under a license for the current study and are therefore not publicly available. However, data are available from the authors upon reasonable request and with permission from the Ethics Committee on Human Research of the Xiangya Hospital Central South University.

## Abbreviations

WBC: White blood cell
Hb: Hemoglobin
PLT: Platelet
ALT: Alanine transaminase
AST: Aspartate transaminase
BUN: Blood urea nitrogen
TBIL: Total bilirubin
DBIL: Direct bilirubin
AMH: Anti-Mullerian hormone
TBA: Total bile acid
CEA: Carcinoembryonic antigen
AFP: Alpha-fetoprotein
CA 15-3: Carbohydrate antigen 15-3
CA 199: Carbohydrate antigen199
CA 125: Cancer antigen 125
CA 72-4: Carbohydrate antigen 72-4
NSE: Neuron-specific enolase
