# Supplementary material for: Intrauterine infusion of clinically graded human umbilical cord-derived mesenchymal stem cells for the treatment of poor healing after uterine injury: a phase I clinical trial
Source: Stem Cell Res Ther. 2022 Mar 3;13:85. doi: 10.1186/s13287-022-02756-9 (PMC8895869; doi:10.1186/s13287-022-02756-9)
Supplement: Supplementary file 1 — Additional file 1. The method of isolation and quality control of clinically graded huMSCs. [file 13287_2022_2756_MOESM1_ESM.docx]

**Materials and methods**

**Isolation and expending of hUC-MSCs**

UC-MSCs were isolated according to a proprietary method^1^. Briefly, Human umbilical cord，from caesarean delivery, immersed in a decontaminating solution, were transported to sterile laboratory by the cold chain transportation within 6 h. The outer amniotic membrane and Wharton's jelly were separated and minced to 1 mm^3^ pieces after washing with D-Hank’s buffer. An optimized precise proportion among tissue mass, enzyme activity units, digestion solution volume and void volume was used for the isolation of cells from the umbilical cord tissue. And the serum-free stepwise culture process was followed growth medium (iSCLCODE^®^). UC-MSCs were plated in culture flask with serum-free medium and placed in a humidified incubator at 37°C, 5% CO_2_ to obtain enough numbers. Cell production was performed in the GMP Cell Production Unit of the SCL now company, with the slight modification of adding support material iECM® hydrogel purified from Wharton’s jelly at protein concentration of 1mg/ml to the final MSCs product.

**Growth curves**

The cells isolated from umbilical cords were seeded in 24-well plates (0.5×10^4^ /well). The number of the cells per well were counted every day for 8 successive days.

**Differentiation of hUC-MSC into Osteocyte and adipocytes in Vitro**

The osteogenic differentiation protection of UC-MSC was assessed in cultures of the third passage ,when the cells were 100% confluent or post confluent. UC-MSCs were seeded in growth medium at 3 × 10^4^ cells/well in 6 P -well tissue culture plates. After 24 hours, the medium was changed to osteoblast Osteogenic Differentiation Medium （HUXUC-90021,OriCell^TM^）. Refeed cells every 3 days by fresh Osteogenic Differentiation Medium. After 2 weeks induction, cells can be fixed and stained with Alizarin red. The osteogenic specific gene, osteopontin (OPN), were further detected by reverse transcriptase polymerase chain reaction (RT-PCR) as described below.

Similar to osteogenic differentiation, the adipogenic differentiation protection of UC-MSCs was also assessed in cultures of the third passage, when the cells were 100% confluent or post confluent. The medium was changed to adipogenic differentiation Medium（HUXUC-90031,OriCell^TM^）A, and Three days later, change the medium to Adipogenic Differentiation Medium B completely for 24 hours . After 3 cycles of Medium A/ Medium B, Fix cells with 2ml of 4% formaldehyde solution and stain with oil red O. *PPAR-r* was selected as the adipogenic specific gene. The appropriate primers are listed in **Table 1**.

**Total RNA extraction and RT-PCR**

By reverse transcription-polymerase chain reaction, the expression of Pluripotent genes *OCT-4、NANOG、SOX-2* and *SSEA-4* was assessed, as well as the reference housekeeping gene b-actin. Total RNA was extracted from 1.5× 10^6^ MSCs cells by using a Total RNA Kit (R6834-01, OMRGA) after cell detachment and washing with PBS. RT-PCR was done following the two-step protocol of the Prime Script™ RT-PCR Kit (RR014A, TAKARA). Primer sets used and the sizes of produced fragments are listed in **Table 1**. Brieﬂy, reverse transcription reactions were performed at 42 ℃ for 30 min hybridization and 70℃for 15 min reverse transcription. PCR reactions were performed at 95 ℃ for 30 s denaturation, primer annealing at 55 ℃ for 30 s; primer extension was performed at 72℃ for 30 s for 30 cycles and 72 ℃ for 10 min as an extra cycle of elongation. Ampliﬁed products were separated on 2% agarose gels containing Goodview for visualization and photographed under UV light. The size of the analyzed genes was estimated by the DNA Ladder (3590A, TAKARA).

**Table1** **Primers used for ampliﬁcation of genes**

| **Primers** | | **Bases Sequence** | **Tm** | **Size** |
| --- | --- | --- | --- | --- |
| *PDX-1*-F | | GTGAGGGAGAAAGATGGACCC | 61.9  60.1 | 335bp |
| *PDX-1*-R | | CAAACATAACCCGAGCACAAGGG |  |  |
| *INSULIN*-F | | ACCAGCATCTGCTCCCTCTA | 59.8 | 115bp |
| *INSULIN*-R | | GGTTCAAGGGCTTTATTCCA | 55.8 |  |
| *NGN-3-F* | | CTATTCTTTTGCGCCGGTAGA | 58.0 | 238bp |
| *NGN-3-R* | | CTCACGGGTCACTTGGACAGT | 61.9 |  |
| *PPAR-γ-F* | | GAAGACCACTCCCACTCCTTTG | 60.0 | 273bp |
| *PPAR-γ-R* | | ATCTCCACAGACACGACATTCA | 56.3 |  |
| *OPN-F* | | CTCCAGTTGTCCCCACAGTAGA | 60.0 | 379bp |
| *OPN-R* | | TGCTCATTGCTCTCATTGG | 56.3 |  |
| *NANOG*-F | CAATGGTGTGACGCAGAAGG | | 59.8  59.8  61.9  57.8 | 94 bp |
| *NANOG*-R | AAGGTTCCCAGTCGGGTTCA | |  |  |
| *OCT-4*- F | GAAGCCTTTCCCCCTGTCTC | |  | 137 bp |
| *OCT-4*- R | AAACCCTGGCACAAACTCCA | |  |  |
| *SOX2*- F | TCAGGAGTTGTCAAGGCAGAG | | 60.0 | 171 bp |
| *SOX2*-R | CGCCGCCGATGATTGTTAT | | 57.6 |  |
| *SSEA-4*- F | ATCTACAACCCAGCCTTCTTC | | 59.8 | 121 bp |
| *SSEA-4*- R | CGTTCACCTCATCACACACAT | | 61.9 |  |
| *β-Actin*-F | CCTAGAAGCATTTGCGGTGG | | 57.5  61.7 | 432 bp |
| *β-Actin*-R | GAGCTACGAGCTGCCTGACGT | |  |  |
| *GAPDH-F* | ACTTTGGTATCGTGGAAGGACT | | 58.2 | 132bp |
| *GAPDH-R* | TAGAGGCAGGGATGATGTTCTG | | 60.1 |  |

**Immunoﬂuorescence**

Cells grown on 24-well tissue culture plates were ﬁxed with 4% paraformaldehyde for 20 min at RT, washed with PBS and permeabilized with 0.25% Triton-X 100 in PBS. After several washes with PBS cells were incubated overnight at RT with the following antibodies against: Rabbit Anti-OCT4 antibody (ab19857, Abcam), Rabbit Anti-SOX2 antibody (ab97959, Abcam) and Rabbit Anti-NANOG antibody (ab21624, Abcam). The corresponding secondary goat anti-rabbit IgG (111-545-003, Jackson) diluted 1:200 in PBS were applied for 2h at RT; mouse Anti-SSEA-4 antibody (Ab16287, Abcam) at a concentration of 15μg/ml. The secondary goat anti-mouse IgG-FITC (Sc-2010, Santa Cruz) were applied for 2 h at RT. After the incubation period the cells were washed extensively in PBST and stained the nucleus with DAPI ([sc-3598](http://www.scbt.com/search/redirect.php?location=datasheet-3598-dapi.html&searchPhrase=dapi&datasheet=sc-3598&tableName=&productType=&page=1), [Santa Cruz](http://www.scbt.com/index.html)) or PI (6607055, Beckman Coulter).

**Flow cytometry analysis**

For ﬂow cytometry analysis, cells were harvested by treatment with 0.125% trypsin (25200056



，GIBCO

), washed with PBS and incubated for 30 min at 4℃ in the dark with the following antibodies purchased from Becton Dickinson and Company: Mouse Anti-Human CD34-PE(550761), CD45-FITC(555482), CD73-PE(550257), CD90-FITC(555595), CD95-PE(555674), CD105(560893), CD29-PE(555443), CD44(550989) , HLA-DR-PE(555561) and HLA-ABC(555552). Rat Anti-Mouse IgG1-PE (550083) and Anti-Mouse IgG1-FITC (553443) were used as negative controls. After that the cells were washed in PBS and 10,000 speciﬁc ﬂuorescence cells was analyzed on FACS (XL, Beckman Coulter).

**Reference**:

1、Han YF, Tao R, Sun TJ, et al. Optimization of human umbilical cord mesenchymal stem cell isolation and culture methods. Cytotechnology. 2013; 65(5):819-827.
