# Supplementary material for: Intrauterine infusion of clinically graded human umbilical cord-derived mesenchymal stem cells for the treatment of poor healing after uterine injury: a phase I clinical trial
Source: Stem Cell Res Ther. 2022 Mar 3;13:85. doi: 10.1186/s13287-022-02756-9 (PMC8895869; doi:10.1186/s13287-022-02756-9)
Supplement: Supplementary file 2 — Additional file 2. The result of isolation and quality control of clinically graded huMSCs. [file 13287_2022_2756_MOESM2_ESM.docx]

**Results**

**Identification and characterization of hUC-MSCs**

After the initial 3 days of primary culture, human MSCs adhered to a plastic surface and presented a small population of single cells with spindle shape. On days 7-10 after initial plating, the cells looked like long spindle-shaped fibroblastic cells, began to form colonies and became confluent. After replating the fibroblast-like cells appeared polygonal or spindly with a long process. The cells were considered normal on the basis of typical morphology. The MSCs were positive for CD73, CD29, CD44, CD105, CD95 and HLA-I but negative for CD34, CD45 and HLA-DR (**Fig.1**).

**Growth characteristics**

The time for 1 passage was about 4-6 days. After the cells were continually passaged for more than 4 months (about 25 passages), they continued to retain their characteristics. The growth curves are shown in **Fig. 2**. The curves of umbilical cord MSCs were ‘‘S’’ type. After re-seeding the cells had 1 day in an adaptive phase. Then the cells began to expand rapidly and move into the logarithmic phase of growth. Six days later, cell counts reached their highest level. According to the growth curve, the population doubling time of the cells was 26 h.

**Pluripotency**

To determine the differentiation potential of UC-MSCs in vitro, we adopted the standard of The International Society for Stem Cell Research (ISSCR).^[[1]](#endnote-0)^ Osteogenic and adipogenic differentiation potential were proved by oil-red O staining (**Fig. 3-E**) and Alizarin red (**Fig. 3-B**) staining respectively. In addition, the differentiation potential of UC-MSCs was proved by specific genes of osteogenic (**Fig. 3-C**) and Adipogenic (**Fig. 3-F**) differentiation marker in mRNA level. Pluripotent regulatory gene detection is another important method for this item. RT-PCR (**Fig. 4**) in mRNA level and immunofluorescence (**Fig. 5**) in protein level was executed independently. The result revealed pluripotency of UC-MSCs in regulatory genes: OCT-4, SOX-2, NANOG and SSEA-4.

1. [↑](#endnote-ref-0)
